# Peripheral and Central Neuroinflammatory Mechanisms Regulating Vagal Sensory Circuit Processing and Cough in Lung Disease

**DOI:** 10.1159/nim/adxag009

**Published:** 2026-09-29

**Authors:** Stuart B. Mazzone, Alexandria K. Driessen, Nathalie A.J. Verzele

**Affiliations:** a Department of Anatomy and Physiology, University of Melbourne, Parkville, VIC, Australia; b Centre of Research Excellence IMPACT Chronic Cough, University of Melbourne, Parkville, VIC, Australia; c Laboratory for Microbiology, Parasitology and Hygiene, Faculty of Pharmaceutical, Biomedical and Veterinary Sciences, University of Antwerp, Antwerp, Belgium

**Keywords:** Vagus nerve, Lung injury, Inflammation, Microglia, Viral infection, Hypersensitivity

## Abstract

**Background:**

The pulmonary system can be exposed to a diverse range of injurious insults which drive pathological events including inflammation, tissue damage, and structural remodelling within the airways and lungs.

**Summary:**

Patients with lung inflammation and injury often experience sensory neurological complications, suggesting that the pathological processes in the lung either impact or extend into the nervous system.

**Key Messages:**

Cough is a common presenting neurological symptom in patients with lung disease and there is growing evidence that neuroinflammation, either within the vagal nerves innervating the airways and lungs or within the central sensory neural circuits regulating cough, is involved in the development of cough disorders. We review and summarise the evidence that lung inflammation and injury drive regional neuroinflammation along peripheral and central vagal sensory pathways, co-ordinated by inflammatory cell recruitment and activation, resulting in altered gene and protein expression, vascular disruption and neurophysiological changes. We also discuss the current therapies in development for chronic cough and highlight the need to consider disease-modifying therapeutics that target neuroinflammation.

## Introduction

Across an individual’s lifespan, their pulmonary system can be exposed to a diverse range of potentially inflammatory and injurious insults, including endogenous pulmonary disease processes or systemic inflammation resultant from other pathological processes, or as a result of exogenous exposures such as viral, bacterial, and fungal pathogens or non-pathogenic agents inhaled from the environment or following aspiration. According to a recent global respiratory health report, more than 80 million people in Europe and as many as 570 million people worldwide are affected by chronic lung disease [1], many of which are characterised by profound pulmonary inflammation that drives widespread tissue damage and structural remodelling, including fibrosis. The underlying pulmonary inflammatory signalling has been studied extensively and shown to involve a wide range of local and recruited cells, inflammatory mediators, and complex regulatory mechanisms. Although some specificity exists in the nature of the inflammation, dependent on the precipitating cause of lung pathology, pro-inflammatory mediators such as tumour necrosis factor alpha (TNFα), interleukin 1-beta (IL-1β), and IL-6 are often stereotypically involved [2, 3].

Lung disease typically presents with respiratory impairment, consistent with acute and chronic respiratory distress, resulting from airway and/or alveolar parenchymal inflammation and damage leading to gas exchange deficits and the ensuing complications of hypoxemia and hypercapnia [4, 5]. In addition to the effects on ventilation, many patients with lung disease experience a suite of sensory-motor neurological manifestations, giving rise to abnormal breathing (breathing pattern disorder), altered perceptions of breathing adequacy (dyspnoea), and excessive coughing [6]. Patients also frequently report cognitive impacts including feelings of “brain fog,” along with mood alterations, lethargy and other changes in behaviour. Indeed, the neurological symptoms in lung disease can be particularly challenging to manage effectively due to a limited understanding of the underlying causative processes, which in turn limits the development of adequate specific therapies. Consequently, these neurological complications profoundly impact an individual’s quality of life, health status and consequently the healthcare system.

In this review, we will focus on the condition of cough hypersensitivity, a particularly troublesome neurological manifestation of lung inflammation and injury [7]. We will specifically assess the evidence that lung disease gives rise to cough hypersensitivity secondary to neuroinflammation along the sensory components of the vagal nerves and central nervous system nuclei involved in regulating cough. We will also briefly discuss some of the emerging therapies in development for targeting the peripheral and central neural elements involved in cough hypersensitivity disorders.

### Acute and Chronic Cough

In humans, cough plays an essential protective role in clearing the respiratory system of a range of materials that are potentially damaging to the airways and lungs, including substances that are locally produced (e.g., mucous) or enter the airways via inhalation (airborne substances in the environment) or aspiration (foodstuffs) [6, 7]. In this function, cough typically proceeds as a reflex mediated by vagal nerves in the airway mucosa detecting the presence of an irritant stimulus and generating a cough respiratory motor pattern by way of inputs to the brainstem respiratory control circuits [8]. Purely reflexive cough is fast and typically under minimal volitional control, and any reduction in the efficacy of reflex cough to clear the airways, such as in patients with spinal cord injuries or following stroke, can lead to potentially catastrophic pulmonary obstructions or infections [9].

Cough is also an extremely common presenting condition accompanying a broad range of diseases. Acute cough is defined as cough persisting for less than 4 weeks and is ubiquitous, with almost everyone in the population experiencing several bouts of acute cough annually due to upper respiratory tract infection (i.e., the common cold) or due to transient environmental exposures to common irritants such as cigarette smoke or other airborne pollutants [10]. The mechanisms leading to acute cough are believed to involve low-grade local airway inflammatory mechanisms, or the direct action of airway irritants, acting upon airway vagal nerves involved in triggering cough [6]. Thus, for most people, acute cough is a self-limiting disorder that resolves spontaneously upon resolution of the underlying infection or removal of the precipitating environmental stimulus [10].

On the other hand, chronic cough is defined as cough persisting for longer than 8 weeks and is a particularly difficult clinical problem to manage [7, 11]. Estimates suggest that between 6 and 10% of the global population suffer from chronic cough [12, 13], and unlike acute cough, chronic cough does not typically resolve without directed intervention [7]. As such, the condition often persists and impacts across several decades of an individual’s life. Furthermore, chronic cough can give rise to secondary manifestations across physical (urinary and bowel incontinence, sleep disturbances, and rib fractures), social (social stigma, embarrassment, withdrawal and isolation, and relationship or work difficulties) and psychological (anxiety, stress, and depression) domains [14–16]. Although chronic cough can be associated with extrapulmonary conditions, such as rhinosinusitis, gastro-oesophageal reflux disease and genetic vagal neuropathies, the most common causes of chronic cough are conditions characterised by pulmonary inflammation and injury [17]. This includes, but is not limited to, conditions such as asthma, chronic obstructive airways disease, idiopathic pulmonary fibrosis and bronchiectasis in which there often exists profound chronic airway and lung inflammation leading to structural pulmonary tissue remodelling and respiratory distress [7]. The mechanical and sheer stresses of repeated coughing may in turn exacerbate an underlying pulmonary pathology, establishing a clinically important perpetual cycle of trauma, injury, and scarring [18].

A near universal feature of patients with chronic cough, regardless of the aetiology, is the existence of cough hypersensitivity, defined as cough evoked by low and often innocuous stimuli [7, 19]. Consequently, patients with chronic cough often experience bouts of debilitating coughing evoked by everyday events and exposures such as talking on the telephone, laughing, the smell of perfumes or eating dry crumbly foods [20]. The existence of cough hypersensitivity, along with accompanying sensations of a persistent tickle or obstruction in the throat, has long been recognised as an indicator that the nervous system involved in regulating cough is abnormally sensitive in patients with chronic cough [7, 11]. However, the mechanisms by which chronic pathologies in the lung lead to neuronal sensitization and chronic cough are unresolved. Notably, although some patients whose chronic cough is associated with an identifiable lung pathology experience clinical improvement in their cough upon effective treatment of their existing lung disease, for many individuals their cough remains refractory to standard disease-modifying interventions [21, 22] suggesting that the mechanisms involved in sustaining altered neural function are unique and require bespoke targeted intervention.

## Peripheral Neurobiology

### Vagal Sensory Innervation of the Lung

The pulmonary system (as shown in Fig. 1) is innervated by sensory neurons whose axons travel mainly within the vagus nerves (reviewed extensively in [6]). Their cell bodies reside outside the respiratory tract in paired bilateral sensory ganglia; the jugular and nodose vagal ganglia. Within these ganglia, neuronal somata (cell bodies) are surrounded by support cells, including immune cells, satellite glial cells, and Schwann cells, which together form an environment that regulates neuronal metabolism, and ion homeostasis [23]. The axons arising from each airway sensory neuron’s cell body splits into two branches, one projection is sent centrally to the brainstem and the other peripherally to the airways and lung. Jugular-derived nerve terminals mostly innervate the extrapulmonary airways, including the larynx and trachea, whereas nodose-derived neurons mainly innervate the intrapulmonary airways and lung parenchyma [6, 24]. The peripheral terminals are found throughout the respiratory tract and terminate within the airway epithelium (including between epithelial cells reaching their apical surface and airway lumen), mucosal glands, smooth muscle, and vasculature. This anatomy places the vagal sensory neurons in an ideal position to continuously monitor the pulmonary environment and initiate appropriate reflex and behavioural responses to salient stimuli [6].

**Fig. 1.**
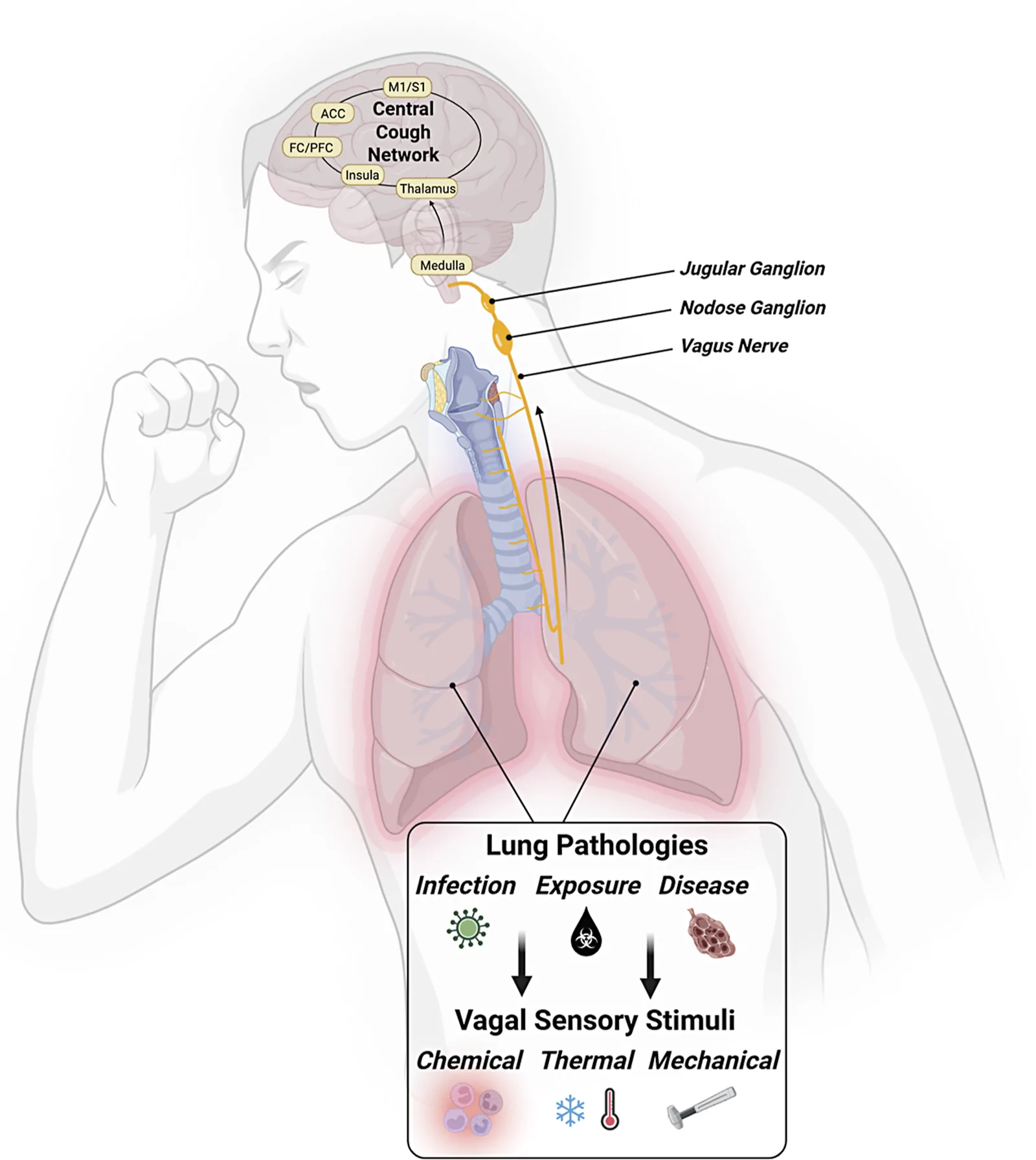
Overview of the vagal neural circuits involved in sensing lung inflammation and injury and regulating cough. The respiratory system can undergo pathological changes due to a variety of pathogenic, non-pathogenic and disease processes. The resultant changes in the chemical, thermal and mechanical microenvironments in the airways and lungs activate nodose and jugular vagal sensory nerve fibres, which in turn provide inputs to brainstem and higher brain circuits involved in generating cough, the accompanying sensations and resultant cognitive impacts. ACC, anterior cingulate cortex; FC/PFC, frontal cortex, prefrontal cortex; M1/S1, primary motor and primary sensory cortex.

The transduction of physical, chemical and thermal stimuli in the airways into neuronal action potentials occurs via a variety of stimulus-specific receptors and ion channels expressed on the peripheral terminals of vagal sensory neurons [6, 24]. With respect to cough and airway defense, as shown in Figure 1 special classes of nodose and jugular vagal sensory neurons termed nociceptors detect noxious exogenous and endogenous stimuli, including the components of inhaled smoke, protons in aspirated gastric contents and a raft of inflammatory mediators released during tissue injury [6, 25]. Nociceptors are frequently characterised by their expression of ion channels of the transient receptor potential (TRP) family. This includes TRP vanilloid 1 (TRPV1), a non-selective cation channel that can be directly activated by noxious heat, protons, capsaicin (from hot chilli peppers), and a range of inflammation-associated lipids [24], or indirectly activated for example through intracellular messengers linked to G-protein coupled receptor (GPCR) signalling [26]. Also, TRP ankyrin 1 (TRPA1), which is directly activated by cannabinoids, nicotine, anaesthetics, prostaglandin metabolites, and molecules associated with oxidative stress [27]. In addition to TRP channels, vagal nociceptors express numerous GPCRs, purinergic (ATP) receptors, and receptors for cytokines (e.g., TNFR1, IL-1βR, interferon [IFN] type I and II) and pathogens (e.g., toll-like receptor 4, TLR4) [6]. Collectively, when activated by a stimulus, these receptors and channels regulate the movement of cations across the nerve terminal membrane, which subsequently depolarises the nociceptor and leads to the generation of action potentials that are conducted centrally to complex neural circuits that ultimately encode cough and related sensations.

### Transcriptional Changes in Vagal Sensory Ganglia in Lung Inflammation and Injury

Many conditions that induce cough are associated with pulmonary inflammation, and as shown in Figure 2 several different preclinical models have shown that accompanying the pulmonary pathology there are also alterations in the transcriptional microenvironment within the vagal sensory ganglia and vagal sensory neurons [28, 29]. In a murine model of asthma induced by exposure to the fungal allergen *Alternia alternata* there was a significant upregulation of genes associated neuronal hypersensitivity, including GPCRs, ion channels and transmembrane ion transport [29]. Surprisingly, however, genes associated with allergen immune sensing were also upregulated in the ganglia, suggesting a level of neuroinflammation accompanying pulmonary inflammation in this model. Consistent with this, pulmonary pathology evoked by infection with influenza A virus was shown to dramatically alter gene expression in both whole vagal ganglia and specifically within vagal sensory neurons innervating the respiratory tract [30]. Of note, many of the upregulated genes were associated with microbial defense and anti-viral responses, suggesting the induction of vagal ganglia signalling responses are an important component of the overall host response to viral infection. Alternatively, vagal ganglia transcriptional changes may reflect a level of nerve injury associated with pulmonary pathology. For example, pulmonary challenge with the bacterial cell wall component lipopolysaccharide resulted in a vagal ganglia transcriptional signature characterised by an upregulation of genes that mediate neuroinflammation, axon growth, and the modulation of airway reflexes [31].

**Fig. 2.**
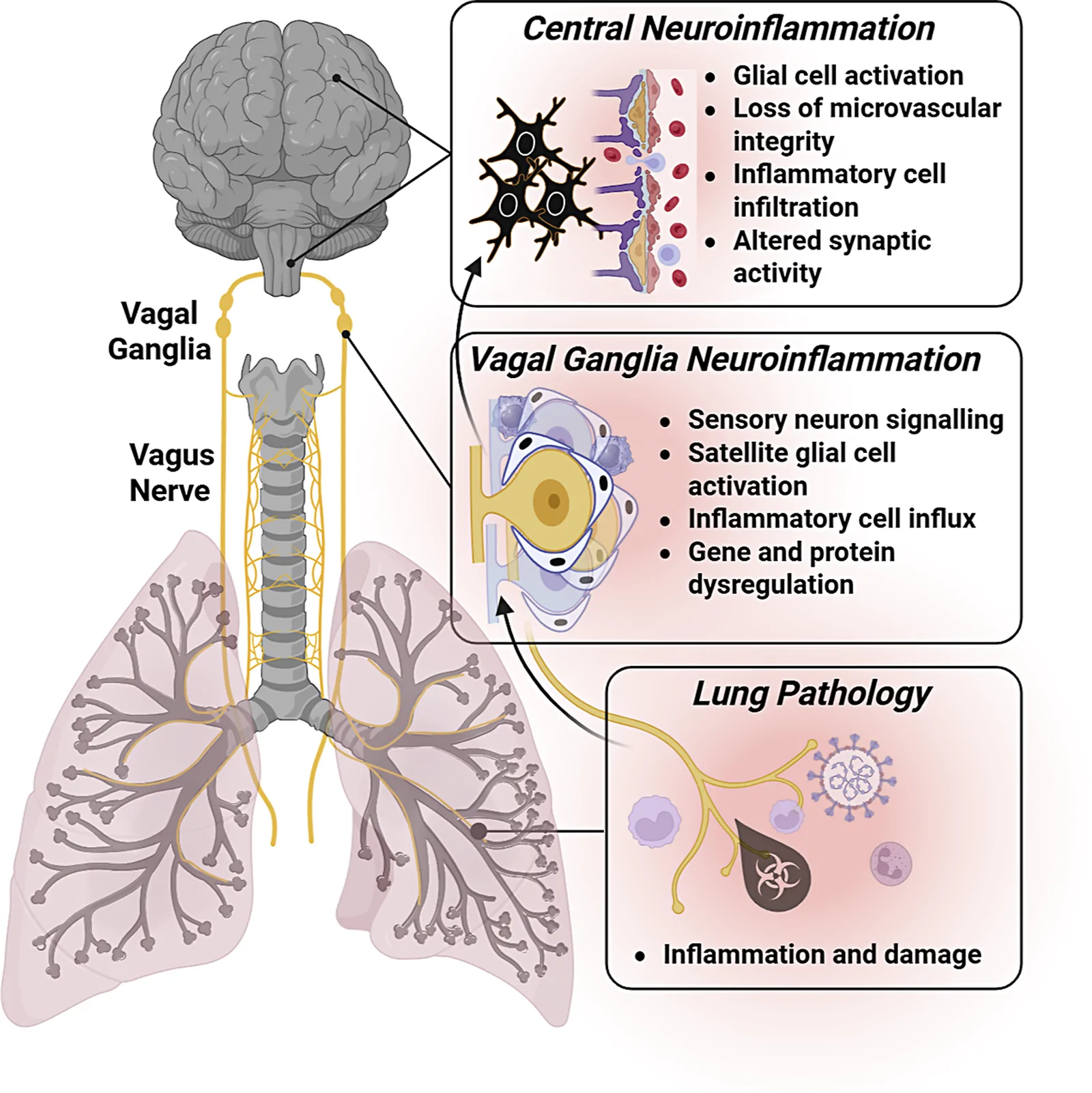
Mechanisms of neuroinflammation in lung inflammation and injury. Vagal ganglia and regions of the central vagal circuitry have been shown to undergo neuroinflammatory changes following induction of lung inflammation and injury. In the ganglia, expansion and/or activation of immune cells and satellite glial cells result in upregulated inflammatory gene and protein expression. In the brainstem, neuroinflammation is characterised by microglial activation the infiltration of systemic immune cells via blood-brain barrier disruption. These processes are likely initiated by signalling along vagal sensory pathways, systemic inflammation or as a result of hypoxia, and collectively the neuroinflammatory events alter sensory neuron and brain circuit neurophysiology leading to establishment of neurological symptoms, such as excessive cough.

Interestingly, pulmonary instillation of inflammatory mediators, including IFN type I, type II, and type III, TNFα, and other viral-induced inflammatory mediators in the absence of active viral infection also induced transcriptional changes, albeit to a lesser extent [30], suggesting that inflammatory signalling itself along vagal pathways (rather than exposure to a pathogen) may be sufficient to initiate neuroinflammatory responses within the vagal ganglia (as shown in Fig. 2). Indeed, a range of cytokines can elicit responses within the sensory neurons. Huerta et al. [32] showed a cytokine-dependent increase in calcium influx in vagal sensory neurons when exposed to IL-10, IL-1β, or TNFα. Importantly, this signalling can occur at the neuronal cell body, but also at the peripheral nerve terminals, as receptors for TNFα and IL-1β are expressed along the length of the vagus nerve as well as within the ganglia [32]. Furthermore, both type I and II IFN receptors have been shown to acutely activate airway-innervating vagal sensory neurons [33, 34], and IFN exposure induces rapid calcium influx, triggering neuronal activation and downstream transcriptional changes. Approximately 70% of TRPV1-expressing (nociceptor) sensory neurons were shown to be stimulated by IFN [33], and IFN exposure in vivo has been shown to result in the development of cough hypersensitivity in guinea pigs and humans [35, 36].

### Immune Cell Changes in the Vagal Ganglia during Lung Inflammation and Injury

Under normal physiological conditions, several immune cell types are detectable in the vagal sensory ganglia, including mast cells and major histocompatibility complex (MHC) class II-positive cells (presumably representing resident tissue macrophages, dendritic cells or perhaps immunological satellite glial cells) [37]. However, the precise role of these cells in homeostatic function, including normal cough reflex regulation, remains to be elucidated. However, they likely play an important role in ongoing immune surveillance, and their activation may contribute to heightened vagal sensory activity and cough hypersensitivity. Notably, several preclinical models have shown an upregulation of vagal ganglia immune cell number or function following induction of pulmonary pathologies known to be associated with cough hypersensitivity (as shown in Fig. 2). For example, MHC class II-positive cell number was significantly increased in the murine vagal ganglia within 6 days following the induction of pulmonary influenza A viral infection, suggesting the establishment of a neuroinflammatory environment with antigen-presenting cell expansion potentially occurring in close proximity to the vagal sensory neuronal somata [30, 38]. This increase in antigen-presenting immune cells during respiratory infection, coupled with the anti-viral transcriptional changes discussed above, might reflect the presence of a pathogenic threat in the ganglia during pulmonary infection, and therefore it may serve to limit pathogen neuroinvasion and replication, as has been shown for other viruses [39, 40]. However, studies using non-infectious pathogen mimetics and antigens, or direct pulmonary instillation of cytokines [30] also report upregulated immune cells in the vagal ganglia, which may indicate that neuroinflammatory signalling along vagal sensory pathways is at least sufficient for vagal ganglia immune cell expansion.

The identity of the immune cells specifically involved in this neuroinflammatory event, the local ganglia signalling mechanisms involved, and the resultant impacts on sensory neural function and cough sensitivity represent important questions under active investigation. Phenotypic characterisation of the upregulated MHC class II-positive cells have identified a possible involvement of vagal ganglia dendritic cells in murine models of house dust mite allergen-induced lung inflammation [37]. However, in influenza viral infection models, whilst MHC class II-positive cell numbers are expanded within the vagal ganglia, dendritic cell and macrophage populations were not significantly increased [30], implying that alternative populations, such as satellite glial cells, may contribute to this response. The origin of the additional immune cells is also unclear as the increase in dendritic cells, following allergic lung inflammation, was shown not to be via locally proliferating cells [37]. Instead, these cells might infiltrate from the peripheral airways via retrograde migration or represent systemic immune cells passing through the ganglion-blood barrier. This suggests that MHC class II-positive cells may have a context-dependent role within the vagal ganglia during pulmonary inflammation and infection. It seems likely, albeit not well studied, that an immunological triad between sensory neurons, classic immune cells and satellite glial cells may be involved in regulating neuroinflammatory events within the vagal sensory ganglia [30, 37, 41, 42]. The nature of the local ganglia signalling events that lead to immune cell expansion may involve molecules released in the ganglia as a result of sensory neuron activation or injury. In support of this, pulmonary viral infection was shown to translocate High Mobility Group Box 1 (HMGB1) from the nucleus to the cytoplasm in vagal sensory neuron somata [43]. HMGB1 is a well-known tissue alarmin, normally located in the nucleus and tightly bound to DNA to stabilise chromatin. However, cell stress and injury can mobilise HMGB1 leading to its release and availability to signal local immune cells via binding to the receptor for advance glycation end-products or TLR4, both of which are also expressed by sensory neurons. These autocrine effects of HMGB1 on sensory neurons may contribute to altered sensory neuron structure and function following respiratory tract viral infections [43–45], perhaps underpinning the development of acute and persistent cough hypersensitivity. Other ganglia mediators released from neurons, immune cells, and satellite glial cells likely include ATP, neuropeptides, cytokines and nitric oxide [41, 46], while more recently highly novel signalling mechanisms have been shown to be involved in the regulation of other neuroinflammatory hypersensitivities, including the physical transfer of mitochondria from satellite glial cells to sensory neurons [47].

## Central Neurobiology

### Overview of Central Circuits Involved in Cough

The central organisation of the circuitry processing vagal sensory inputs and regulating coughing is complex (as shown in Fig. 1), and a full description is beyond the scope of this review. A brief overview of the basic central neuroanatomical framework with respect to cough processing is provided, and readers seeking more detailed information are referred to comprehensive reviews previously published [7, 48]. An important point to note is that although subsets of vagal sensory neurons from both the nodose and jugular ganglia regulate cough through inputs to medullary brainstem circuits, the central termination patterns of these different populations of vagal sensory neurons are quite distinct and non-overlapping [49]. Nodose neurons involved in cough and respiratory reflex regulation terminate centrally in the caudal regions of the nucleus of the solitary tract, whereas jugular neurons terminate in and around the paratrigeminal nucleus [49]. This differential organisation pattern has been demonstrated in rodents using neuroanatomical tract tracing methods and subsequently confirmed in humans using functional brain imaging [49–51]. In turn, the nucleus of the solitary tract and paratrigeminal neurons in receipt of primary vagal sensory inputs connect to pontomedullary circuits involved in respiratory and autonomic control, via which vagal inputs can regulate breathing motor patterns to generate reflex coughing, apnoea and other defensive respiratory responses [6, 52]. In addition, projections from the nucleus of the solitary tract and paratrigeminal nucleus ascend the neuraxis, albeit through differing circuits, to terminate in thalamic and other subcortical forebrain regions [49]. Outputs from these sites in turn contribute to distributed cortical higher brain networks important for perceiving airway sensations and generating cognitive and behavioural responses [53, 54], including through descending neural pathways that directly influence vagal sensory processing in the lower brainstem medullary nuclei [55]. These complex ascending and descending circuits connecting the vagal sensory integration sites in the medullary brainstem to the higher brain networks have recently been the subject of much interest, as changes in the efficacy of neural processing at locations along these networks is believed to be important in the development of cough hypersensitivity [51, 56].

### Central Manifestations of Neuroinflammation in Lung Inflammation and Injury

An important question is whether pathogenic or non-pathogenic lung inflammation and injury is accompanied by neuroinflammation extending into central nervous system, thereby representing an alternative mechanism underlying the development of disordered cough and other neurological impacts. In this regard, several studies have provided evidence supporting the establishment of central neuroinflammation following the induction of lung disease. While a variety of different brain regions have been investigated, structural and/or functional neuroinflammatory changes in the nucleus of the solitary tract, hippocampus, amygdala and across the cerebral cortex appear to be the most commonly identified sites impacted [57–64]. In such cases, as shown in Figure 2, regionalised activation of microglia and astrocytes, altered cytokine expression, blood-brain barrier dysfunction, and leukocyte trafficking into the CNS occur, leading to neuronal dysfunction impacting discrete brain networks. For example, preclinical studies employing influenza viral infections [64], pulmonary exposures to bleomycin or allergen sensitization and challenge models [57, 58, 60], including for the induction of asthma [65] have all shown increased glial activity, particularly in the nucleus of the solitary tract, hippocampus and the amygdala. Notably, glial cell activation is accompanied by upregulated cytokine and chemokine expression, with IL-1β, IL-6, TNFα, and the chemokine CCl2 representing the most commonly implicated mediators orchestrating central neuroinflammation. However, many other mediators may also be involved, including reactive oxygen species, nitric oxide, NOD-like receptor signalling (i.e., the NLRP3 inflammasome), JAK-STAT signalling, and macrophage anti-inflammatory pathways [64, 66].

During pulmonary viral infections, central neuroinflammation appears to occur despite little or no direct evidence for any direct inflammatory effects due to viral invasion into the brain [61, 62, 67]. Similarly, bleomycin, lipopolysaccharide, allergens and other mediators of pulmonary injury are not believed to directly access the central nervous system. This implies that peripheral signalling must play an important role in the establishment of central neuroinflammation and several likely causative mechanisms have been identified. A common and important finding associated with lung disease is a reduction of blood-brain barrier integrity [64, 66, 68]. This effect is believed to be mediated by circulating inflammatory mediators acting on cerebral vasculature endothelial cells, promoting altered gene and protein expression (including β-actin and adhesion molecule expression), increasing oxidative stress, and enhancing matrix metalloproteinase activity (*MMP1, MMP3, and MMP16*). These cumulative effects disrupt the tight junctions that normally maintain the blood-brain barrier [69]. Given that the blood-brain barrier normally plays an important role in limiting the movement of cells, pathogens and chemicals between the vasculature and brain parenchyma, a reduction in barrier integrity increases the exposure of the brain to systemic inflammation, which often accompanies lung pathologies. Subsequently, infiltrating circulating inflammatory cells can access the brain parenchyma and therefore act in concert with local glial cells to amplify central neuroinflammatory events. In addition, systemic hypoxemia and tissue hypoxia associated with poor ventilation may further compound central inflammatory injury [69]. Persistent vagal sensory neuron activation, expected as a result of the pulmonary inflammation associated with lung pathology, may also serve to promote central neuroinflammation as sensory neuropeptides and other neurotransmitters can spillover from central synapses and directly activate local microglia and astrocytes [70].

The functional consequences of central neuroinflammation have not been extensively studied but accompanying changes in regional neuronal activity have been reported. This has been best studied in the nucleus of the solitary tract, although other regional changes in neuronal activity have also been reported [60, 65, 71–75]. Neurons impacted by neuroinflammation express increased reactive oxygen species, perhaps indicative of elevated neuronal stress, and undergo a variety of changes in gene and protein expression, including alterations in Na^+^K^+^-ATPase and membrane ion channels needed to regulate normal resting membrane potential [76]. Electrophysiological studies demonstrate both increased and decreased excitability of local neural circuits in the nucleus of the solitary tract, depending on the nature of the lung disease under investigation. For example, following allergic or cigarette smoke-induced lung inflammation, nucleus of the solitary tract neurons have reduced membrane potentials, enhanced responses to vagal afferent stimulation, and increased glutamatergic neurotransmission, which in part may be due to reduced local inhibitory neurotransmission [72, 73, 75]. By contrast, lung exposure to bleomycin has been shown to reduce synaptic efficacy between vagal sensory inputs and second-order neurons in the nucleus of the solitary tract, an effect related to altered expression of excitatory glutamate receptors [74]. Overall, these functional impacts on local circuit activity likely underpin the neurological symptoms that arise in lung pathologies, although more research is needed to understand these central functional processes in greater detail. Patients with chronic cough demonstrate altered central cough network structure and function [51, 56, 77], although whether this is a result of central neuroinflammation is not known.

### Targeting Vagal Pathways to Treat Cough

There has long been interest in identifying therapies that target peripheral or central components of the vagal sensory nervous system to deliver clinical benefit in pulmonary disease, especially to identify novel therapies to treat chronic cough. Many original efforts focussed on sensory neuropeptide signalling, including drugs that inhibit the peripheral or central neurokinin 1 receptors for substance P [78]. This rationale appeared reasonable given that preclinical rodent studies identified significant involvement of neuropeptides in vagal-induced neurogenic inflammation and the central signalling of sensory processing [78], although clinical trials of drugs assessing neurokinin 1 receptor antagonists in patients with chronic cough have largely been disappointing [79]. Notably, substance P can also bind to other receptors, including the Mas-Related G Protein-Coupled Receptors MrgprA1 and MRGPRX2 (or the murine ortholog MrgprB2), expressed by sensory neurons and mast cells in the airways, perhaps offering an alternative neuroinflammatory signalling pathway mediating the actions of substance P [80, 81]. However, to date there have been no clinical trials targeting these receptors in chronic cough. Nevertheless, it is worth recognising that the evidence for neuropeptide expression in human vagal sensory neurons is much less compelling than it is in rodents, suggesting substance P may not be a critical player in airway neuroimmune interactions in humans [78]. A significant preclinical effort has also been focussed on identifying receptors and ion channels expressed by vagal nociceptive sensory neurons and shown to be activated by lung inflammation [6, 25, 26, 34]. Sensory neuron TRP channels were particularly exciting candidate targets, again with preclinical rodent studies demonstrating benefit of inhibiting TRPV1, TRPV4, or TRPA1 in various models of lung inflammation and injury [82]. However, human antitussive clinical trials assessing drugs acting at each of these channels have also been disappointingly negative [22].

More recently, attention has turned to purinergic signalling in the lung, given a demonstrable role of extracellular ATP, released from activated or injured airway mucosal cells, acting upon vagal sensory neuron P2X3 and P2X2/3 receptors [6, 7, 22, 24]. Several clinical trials have assessed selective P2X3 antagonists in patients with refractory chronic cough, albeit with mixed success. One P2X3 antagonist, gefapixant, has made it to market in Japan and Europe while another, camlipixant, recently stumbled at late-stage phase III trials, failing to meet primary endpoint efficacy in one of the two parallel global trials [83, 84]. Several other companies have ceased in their P2X3 antitussive development programs either due to lack of efficacy or unfavourable off-target effects [85, 86]. An alternative strategy in development is to inhibit pulmonary vagal sensory neurons more indiscriminately by targeting the sodium channels involved in vagal sensory action potential generation. In this way, vagal sensory inhibition (local anaesthesia) can be achieved irrespective of the inflammatory drivers present in the lung. Sensory neurons uniquely rely on the NaV1.7, NaV1.8, and NaV1.9 subtypes of the voltage-gated sodium channels, and a novel NaV-selective anaesthetic-like drug formulation (delivered through a dry powder inhaler to limit exposure to the airways) has recently completed phase II trials for chronic cough [22, 87], and resultant outcomes are pending release.

In clinical practice, currently available centrally acting neuromodulator drugs, including gabapentin, pregabalin, amitriptyline and baclofen, are commonly trialled in patients with refractory chronic cough [22, 88]. The therapeutic efficacy of these central neuromodulators is presumed to be a result of targeting central mechanisms driving hypersensitivity, akin to that in patients with chronic neuropathic pain in which central neuromodulators are also often effective. Nevertheless, patient responsivity and tolerability to these agents is heterogeneous, which limits their use [88]. Similarly, low-dose morphine (or other narcotic opiates) remains a clinical option in patients with difficult to manage chronic cough, with recent evidence suggesting that they specifically target abnormal sensory processing in the brain [88, 89]. Several recent trials have also advanced novel centrally acting antitussive drugs with fewer off-target effects. For example, an extended-release oral formulation of the kappa-opioid receptor agonist/mu-opioid receptor antagonist nalbuphine is reasonably well-tolerated and shown to produce robust suppression of chronic cough, including in patients with pulmonary fibrosis [90]. Allosteric modulators of glutamateric N-Methyl-d-Aspartate receptors and GABAergic receptors are also in current development although clinical trial data is limited. Further studies are needed to identify other central druggable processes for therapeutic development.

The fact that the last FDA-approved drug for treating cough, dextromethorphan, was approved in 1958, along with the growing list of disappointing antitussive clinical trial outcomes, suggests a challenge exists in translating our detailed mechanistic knowledge obtained from preclinical studies into effective therapies in clinic. The reason for this is likely multifactorial across both trial design (including the choice of end-point measures and larger than expected placebo responses in antitussive trials, reviewed elsewhere [91, 92]) and biological considerations. With respect to the latter, although chronic cough is common across many conditions, it is unlikely that the underpinning neuroinflammatory processes are homogeneous. Instead, distinct cough endotypes likely exist, representing the unique peripheral and/or central pathological processes that contribute to the development of cough hypersensitivity [93]. At present, effective tools such as chemical or genetic biomarkers or clinical phenotyping tests for stratifying patients into appropriate clinical cohorts are lacking. Consequently, clinical trial cohorts may not be optimised for the mode of drug action. This was evident in P2X3 trials where less than 50% of trial participants were proven to be responders, diluting the overall placebo-adjusted efficacy of gefapixant [83], and is not dissimilar to the challenges that biologic agents faced in asthma clinical trials several decades ago [94]. Additionally, for the current suite of drugs under investigation, there is no clinical or preclinical evidence to suggest that any modulate peripheral or central neuroinflammation that likely contributes to the cough disorder. As such, these therapies may not prove to be disease-modifying, instead only delivering on-drug symptomatic relief. Similarly, whether the therapies showing promise for alleviating chronic cough will more broadly be beneficial by altering other neurological complications accompanying lung inflammation and injury is not known and this may impact overall patient impression of treatment or quality of life measures. Studies addressing these important considerations have not been conducted, representing important gaps in our current knowledge which might help improve clinical translation in the future.

Non-pharmacological (behavioural) therapies are also commonly trailed in patients with persistent chronic cough. Such therapies deliver a combination of speech and language therapy centred around modified breathing techniques, cognitive behavioural therapy, laryngeal hygiene and chronic cough education, and are often effective for patients who persist with therapy. The mechanism of action of these therapies is unknown, although they may help retrain patient perceptual tolerance of airway irritation or improve cough suppression strategies through higher brain mechanisms [95]. Whether such therapies modify neuroinflammation is not known.

## Conclusion

Neurological complications, including chronic cough and other sensory and behavioural manifestations, are common in patients with lung inflammation and injury, and their management proves challenging. Extensive preclinical evidence supports the notion that pathologies impacting the lung also promote inflammation within the vagal nerves and along the central circuits involved in vagal sensory processing. This neuroinflammation alters peripheral and central neuronal activity and is therefore likely important in the development and maintenance of chronic cough and related neurological symptoms in lung disease. Current therapies are in development for managing chronic cough, mainly targeting aberrant neural activity along peripheral and central vagal pathways. However, as the pathological processes differ across different lung diseases, whether this strategy will remedy the potentially heterogenous modes of neuroinflammation that can conceivably lead to aberrant vagal sensory circuit neural activity remains to be seen. Accordingly, opportunities exist for developing drugs that target defined neuroinflammatory processes and arrest the problem at its source.

## Conflict of Interest Statement

S.B.M. has received consulting fees from GlaxoSmithKline (G.S.K.), Merck and Co, NeRRe Therapeutics, Reckitt Benckiser, Chiesi Farmaceutici, iNOVA, and Trevi Therapeutics, Inc.; serves on advisory boards for Reckitt Benckiser, G.S.K., Trevi Therapeutics and Seyltx; and holds grant support from Merck & Co and Reckitt Benckiser, unrelated to the content of this review. The remaining authors have no conflicts of interest to declare.

## Funding Sources

This work was supported by the National Health and Medical Research Council of Australia [Grants Nos #2018988 and #2044698]. The funder had no role in the design, data collection, data analysis, and reporting of this study.

## Author Contributions

S.B.M. conceived the topic and scope of the review. S.B.M., A.K.D., and N.V. contributed equally by drafting different sections of the manuscript (including figures). S.B.M. compiled and assimilated each author’s contribution into a complete draft that was then reviewed and edited by A.K.D. and N.V. All authors approved the final submission.
